# Computer-Mediated Communication and Child/Adolescent Friendship Quality after Residential Relocation

**DOI:** 10.1007/s10826-021-02102-2

**Published:** 2021-09-18

**Authors:** Ruth Wendt, Alexandra N. Langmeyer

**Affiliations:** 1 Leibniz-Institut für Wissensmedien (Knowledge Media Research Center), Schleichstrasse 6, 72076 Tuebingen, Germany; 2 Deutsches Jugendinstitut (German Youth Institute), Nockherbergstrasse 2, 81541 Munich, Germany

**Keywords:** Computer-mediated communication, Face-to-face communication, Friendships, Children, Relocation

## Abstract

The experience of residential relocation can affect children and adolescents in various ways. It often affects their close social relationships, and this is especially true when these individuals are no longer in close proximity to their family members and friends. Although face-to-face communication may be limited after relocation, computer-mediated communication can assist in maintaining and developing existing relationships. It may even help individuals initiate new social relationships. In the present study, we investigated the role of communication behavior with friends for perceived friendship quality among children and adolescents who recently experienced residential relocation. Based on a representative survey study of families in Germany, we selected parents having moved with their child (8 to 14 years) to another village or town within the last 24 months. In total, 57 parents who had recently moved – majority of whom were mothers – allowed their child to participate in the phone interview. These participants were, on average, 11 years of age, and 58% of them were male. The children answered questions about their communication behavior and the friendships they had with their three current best friends. Using multilevel analysis, we found that children and adolescents who more often communicated face-to-face with their friends also more intensively used computer-mediated technologies to stay in touch. However, the findings further revealed that computer-mediated communication has the potential to remove limitations in existing friendships in the event of relocation. In the following discussion, implications are examined regarding the role of new communication technologies for families with enhanced mobility requirements.

## Highlights

- Children & adolescents used social networks to stay in touch with close friends after moving into another town.
- Increased face-to-face communication with friends was directly related to increased communication via computer-mediated technologies.
- When children & adolescents communicated with a friend via social networks, they reported a higher friendship quality.
- Quality of computer-mediated communication – and not the intensity thereof – positively influenced the perceived friendship quality.

## Introduction

Digital media permeate all areas of our life and influence the basic structures of our interpersonal communication. Nowadays, a large part of our everyday communication - whether private or professional – is computer-mediated. Mobile media have developed into multifunctional everyday companions (Döring, 2014) that offer a multitude of communication occasions and possibilities. People think, feel, experience, and act with the expectation of being permanently online and permanently connected (Vorderer, 2015). This aspect particularly shapes social relationships. Constant availability not only transcends geographic boundaries between persons, but also shapes the communication culture of those who are located in the same place (e.g., within families). Regular face-to-face (FtF)-communication is supplemented by a plethora of short calls or messages via various platforms, and this strengthens the everyday co-presence of social relationships (Utz, 2017). Computer-mediated communication (CMC), however, can especially enable frequent contact with family members or friends who are no longer living in the immediate proximity.

Due to different socio-economic developments, children and adolescents are increasingly confronted with spatial mobility requirements. This can be explained from a variety of standpoints. The first of these standpoints lies in the economic factor. Due to globalization processes and an increased number of working women (and specifically women with children), the job market demands a flexible deployment of skilled workers at changing places of work (Schneider et al., 2014). Recent studies, however, have confirmed that an increase in occupational mobility experiences is exclusively due to an increase in circular mobility (e.g., long-distance commuting), while the number of long-distance removals for occupational reasons has remained constant (Schneider et al., 2014). Family status – and the changes thereof – is the second factor that residential relocation during childhood can be attributed to (see Anderson et al., 2014a; 2014b). Feijten and van Ham (2013) found that the separation or divorce of parents is often accompanied by more frequent relocations. However, the distance between the parent with children (typically the mother) and the parent without children is generally rather short. In addition to change of residence, children from separation and divorce families are often confronted with a multilocal lifestyle. Data from a representative survey study in Germany confirmed that 13% of underage children have separated parents. Of this percentage of children, over 60 percent live alternately with both parents (Schier, 2013).

Independent of the reasons, child and adolescent residential relocation is assumed to affect their social relationships. This is especially true in the event that family members and friends are no longer living in the immediate proximity. Although FtF-communication can potentially be limited after moving away, it is assumed that CMC is not affected by geographical boundaries. In fact, it may even aid in maintaining existing social relationships and initiating new ones. Previous research particularly confirms that social media use positively influences friendship closeness among children and adolescents (for an overview see Pouwels et al., 2021). In the present study, we thus focused on friendships and analyzed the role of child and adolescent communication behavior (both FtF and computer-mediated) with friends. The aim of this research was to examine the participants’ perceived friendship quality after experiencing residential relocation, describing a specific and stressful living situation that comes along with certain social cutbacks.

### Residential Relocation and Social Relationships

Several empirical studies have previously confirmed the effects of residential relocation on the health, psychosocial development (e.g., victimization or deviant behavior), and school performance of children (see Humke & Schaefer, 1995; Jelleyman & Spencer, 2008), and the majority of these effects were negative. An intensification or development of behavioral problems after a change of residence was especially found for children in early adolescence (Wood et al.,^ 1993^). Considering the detrimental effects, it must be specified which underlying mechanisms associated with the relocation of children and adolescents negatively influences their development.

By combining bio-ecological approaches and a developmental system perspective, Anderson and colleagues developed a framework model that can be used to locate the significance of residential relocation experiences for the growth and development of children (Anderson et al., 2014a; 2014b). According to the model, changes caused by relocation primarily affect the immediate social contexts that children are embedded in and within which they interact on a daily basis. These primarily consist of the family and the peer contexts; however, the school and the neighborhood are also included. Relocation mostly affects changes in these social contexts, as it requires a reorganization of the social environment. As previously claimed, residential relocation of children and adolescents often goes hand-in-hand with a change in family structure. It may even be triggered by this change. However, even if family constellations remain the same, a change of residence can also have an influence on family processes and – via these – on the social life of children. For parents, relocation is often associated with further stressors, such as additional financial burdens, occupational changes, or even the loss of social resources. It is expected that such changes – temporarily can influence the climate and relationships within the family (see Anderson et al., 2014a; 2014b).

In addition to this socio-ecological perspective, a developmental dimension must be considered when describing the influence of certain transitions and life events within the individual developmental phases of children (Bronfenbrenner & Morris, 2006). When transiting from childhood to adolescence, children increasingly emancipate themselves from their parents, which is a balancing act between parental control and youth autonomy (Seiffge-Krenke, 1997). On the other hand, the relevance of orientation towards peers – whose appreciation and recognition are central to building a positive sense of self-worth – is intensifying (Oswald, 2009). Previous research has confirmed that peer influence has a considerable effect on numerous attitudes and behaviors of children and adolescents (e.g., Bukowski et al., 2007). Reitz et al. (2014) have also emphasized that, in addition to peer norms that are frequently analyzed in previous studies, the dyadic friendship relationships embedded in larger peer groups also play an important role in child development.

Depending on the distance, a change of residence is often associated with a deterioration in both the number and quality of peer relationships. This comes as a result of leaving existing friends and acquaintances behind. However, many findings suggest that perceived limitations in peer relationships can be compensated by building up new friendships after a certain period of time (e.g., Vernberg et al., 2006). Despite this fact, many children and adolescents find it challenging to build up new friendships (see Vernberg & Randall, 1997). Studies, for example, have shown links between the residential relocation of children and their deviant behavior mediated by negative aspects of their peer relationships, including a higher number of deviant peers and smaller, less popular peer networks (e.g., Haynie & South, 2005). However, other researchers suggest that differences may have previously existed within peer relationships between mobile and spatially stable children and adolescents (Gasper et al., 2012). Nevertheless, these findings show that entering new peer groups and building new friendships is challenging for many youngsters, and it may initially result in specific peer constellations. It also becomes clear that peer relationships play an important role in the psychosocial development of children, although the focus has rarely been directed towards qualitative aspects of social relationships (see Anderson et al., 2014a; 2014b).

In summary, residential relocation of children and adolescents is primarily associated with short-term influences on their immediate social contexts, which in turn can explain changes in their psychosocial development. Particularly among older children, peer relationships become increasingly important and are assumed to be restricted and interrupted after a child’s change of residence. In the present study, we thus focused on child and adolescent friendship quality after experiencing a recent relocation.

### The Moderating Role of CMC

Regular communication was found to be crucial for the building, developing, and maintaining of social relationships (see Vangelisti, 2015), especially regarding the more fragile friendships of the childhood years (Gifford-Smith & Brownell, 2003). Expected restrictions in these after a change of residence are primarily attributed to a loss of everyday direct communication. However, CMC modes nowadays enable users to permanently stay in touch, multimodally interact, and communicate 24/7 without being located in the same place. Since the emergence of CMC, several theories have arisen comparing users’ responses and adaption to CMC as compared to FtF-communication. A very established strain of research, referred to as “cues-filtered-out theories” (Culnan & Markus, 1987), argued that CMC is systematically reduced by nonverbal cues, resulting in more impersonal orientations among users. In contrast, other theories emphasized the complexity and the potential of different media, arguing that users can adapt to these cue limitations and achieve a somewhat face-to-face level of affinity, depending on user characteristics as well as social and contextual factors (Walther, 2011, p. 444–445).

In the context of peer relationships, the use of CMC is especially preferred among children and adolescents. Usage data confirm that a large part of youngsters’ online use describes their engagement in communication with others (Medienpädagogischer Forschungsverbund Südwest, 2020). Many adolescents have made new friends online, and nearly all of these individuals use social media to stay in contact and maintain daily interaction with their friends (Lenhart, 2016; Rideout & Robb, 2018; van Driel et al., 2019). CMC ensures a permanent contact with peers and prevents children and adolescents from missing out (Hefner et al., 2018). In addition, it facilitates the everyday organization of friendship activities and enables direct and immediate contact without having to go through parents (Knop et al., 2015). Finally, it also helps young users to better understand their friends, and this stimulates a feeling of close association (Borca et al., 2015). To sum up, CMC opens new communication spaces for children and adolescents and offers a wide range of options for dealing with relationship-relevant topics and needs (Schulz, 2013).

Accordingly, CMC can be an important resource in the face of discontinuities that children and adolescents experience in their social environment. It provides opportunities to make new friends or stay in touch with existing friends regardless of a user’s location. Previous studies have primarily focused on stationary stays of children and adolescents due to illness. Van der Velden and El Emam (2013), for example, have shown that young patients in stationary facilities primarily use Facebook to continue participating in their regular social life with friends. According to these results, most youngsters rarely use social media to contact other people with a similar living circumstance. Schulz (2013) refers to the central role of the mobile phone for children and adolescents who grow up with a locally separated parent. Accordingly, individualized mobile communication makes it possible to establish contact independently, and it promotes the reintegration of the separated parent into the child’s everyday life. Thus, CMC can buffer social cutbacks after residential relocation. However, underlying mechanisms and dynamics have hardly been investigated so far, especially when it comes to child and adolescent friendship relationships.

### The Present Study

The main objective of the present study was to investigate the role of child and adolescent communication behavior with friends for their perceived friendship quality after experiencing residential relocation and describing a specific, stressful living situation that might be accompanied by certain social cutbacks. First, it can be assumed that a young users’ communication behavior with friends may differ after a change of residence, depending on certain relocation-related aspects (e.g., the living distance between the friends). In a first research question, we therefore asked the following:

RQ1: How does residential relocation influence the FtF-communication and CMC of children and adolescents with their close friends?

Previous research has confirmed that nearly all children and adolescents use CMC to stay in contact and connect with their peers (Lenhart, 2016; Rideout & Robb, 2018; van Driel et al., 2019). For this reason, this mode of communication is potentially and particularly significant when children and adolescents experience a move away from their friends. We, therefore, asked the following:

RQ2: Can CMC buffer the negative influences of residential relocation on perceived friendship quality with close friends who users knew prior to the relocation?

Previous research also exhibited that a certain number of youngsters also use CMC to initiate new friendships (Lenhart, 2016). Thus, CMC may be one important way for young users to make new friends following a relocation, and it may also aid in consolidating newly made friendships that, as an example, were first initiated in the new school. We therefore asked:

RQ3: Can CMC strengthen the perceived quality for new friendships initiated after the residential relocation?

Finally, research on interpersonal communication behavior has previously confirmed that both frequency and quality indicators of communication shape the perceived quality of close social relationships (Emmers-Sommer, 2004). To our knowledge, the quality of CMC in child and adolescent friendships was not previously analyzed in a systematical manner. In a final step, we therefore asked:

RQ4: Does the perceived quality of CMC with close friends influence the overall friendship quality?

## Method

### Participants & Procedure

The present research was part of a large-scale, representative survey study of parents in Germany. This study investigated the care situation regarding one target child aged between 0 and 14 years (*N* = 54.064; KiBS, Alt et al., 2017). Data were drawn by a professional market research institute that recruited target children via registration offices, and this equally covered all German federal states despite disproportionally covering the various age groups (u3, u6, u11, and u15). The KiBS-study was conducted at the German Youth Institute in 2016 and 2017 and was funded by the German Federal Ministry of Family Affairs, Senior Citizens, Women, and Youth. In the study, the main caring parent (which – in most cases – were the mothers) answered a standardized telephone interview, online survey, or paper-and-pencil questionnaire about the respective target child. This method mix was chosen to maximize the reachability of the target sample. Every respondent could select the preferred format of the survey. For the present study, a filter question was integrated in this survey, asking the parent of target children between 8 and 14 years (*n* = 19.629) if she or he had moved to another town or village with the child within the last year (*n* = 518, 2,6%). If the parent agreed, he or she was asked to participate in an additional standardized telephone survey on digital media and communication. This additional study included questions for the parent regarding general information on the last residential relocation. It also included questions for the target child regarding specific information on their media use and communication behavior with their parents and friends. This study on digital media and communication – funded by the German Youth Institute – collected data from January 2017 to November 2017. Of the 518 parents fulfilling the selection criteria, 79 parents agreed to participate in the study (15%). Only 57 parents, however, also allowed the subsequent child interview (11%). No significant differences were found between the dropouts (*n* = 461) and the final sample (*n* = 57) regarding gender and age of the child, as well as their socioeconomic status indicated by net household income and the highest level of education per household.

For the main analyses, the final sample of 57 respondents was used. In most cases, the biological mothers of the children answered the questionnaire (95%, *n* = 54), while the remaining 3 informants were the biological fathers. The children on average were 11 years of age (SD = 2.0) and 58% were males (*n* = 33). The mean net household income of the families (weighted according to the OECD scale) was 1859€ per month (*SD* = 838, min = 385, max = 3913). Furthermore, most of the children were living in a high-educated household (68%; 4% lower-educated, 28% medium-educated). Finally, a large part of the children indicated use of the Internet via their own smartphones (*n* = 40), computer/laptops (*n* = 20), tablets (*n* = 18), or gaming consoles (*n* = 13).

The residential relocations of the families on average took place 12 months prior (SD = 6.5; min = 0; max = 24). Since it is a rarity to find families with school-age children who have recently moved, the data collection of the project lasted for several months. The average time since relocation was comparably large, and this was due to different time points of the family interviews over the course of a 10-month data collection period. Some families (*n* = 11) stayed in the same neighborhood, village, or town. A larger part (*n* = 26) had moved to another village or town no more than an hour away from their previous location. Nearly one third of the families (*n* = 17) had moved further away in Germany, or even abroad. Three families did not answer this question. Regarding the different reasons for their relocation, 21 families mentioned “occupation/education/studies”, followed by “family” (*n* = 10) and “improvement of the residential area” (*n* = 10). A smaller part of eight families place the reason for their relocation on separation, divorce, or a new partnership. More than half of the children (*n* = 29) changed schools after their family’s relocation. Finally, parents indicated an average of 3 relocations they had already made with their target child (*M* = 3.2; SD = 3.0; min = 1; max = 17). Neither the net household income per month (*r* = 0.02, *p* = 0.864) nor the highest level of education per household (*r* = 0.11, *p* = 0.446) significantly correlated with the number of residential relocations in the families.

### Measures

The following measures were all answered by the target children themselves.

#### Friend

We asked the children and adolescents to name their best friends by their first name, allowing for up to three nominations per child. If the child did not initially mention a name, the interviewer would ask once more to ensure whether or not there was anyone the child wanted to mention. Following this procedure, every child mentioned at least one friend. In total, the children and adolescents named 149 friends (2.6 friends per child).

#### Friendship initiation

For each of the nominated friends, we asked the child if she or he got to know this friend before (0) or after (1) his or her last residential relocation. In total, 43 percent (*n* = 63) of the friends were met after the last relocation.

#### Friend’s living distance

To measure the respective living distance between the child and his or her friends, we asked where each of their nominated friends lived. The child answered this question for each friend on a 6-point scale, ranging from 1 (“in the same house”), 2 (“in the neighborhood”), 3 (“in the same village/town, but more than a 15 min walk”), 4 (“in another village/town, but reachable in one hour”), 5 (“further away in Germany”), or 6 (“further away abroad”; *M* = 3.2, SD = 0.9). The response format was adopted from the German survey “Growing up in Germany” (Walper et al., 2015). In total, 41 percent (*n* = 60) of the friends lived in a different village or town than the respondents.

#### Communication behavior with friends

Regarding communication behavior with friends, we differentiated between FtF-communication and CMC. The former was measured by asking the child how often she or he sees or meets each of the nominated friends. The response format ranged between 0 (“never”), 1 (“more seldom”), 2 (“at least once a month”), 3 (“once a week”), 4 (“several times a week”), and 5 (“every day”; *M* = 3.7, SD = 1.3). To measure CMC, the child indicated how often she or he has contact with each of the nominated friends via “*telephone*” (*M* = 2.4, SD = 1.7), “*text messages on the phone* (e.g., WhatsApp, Snapchat, SMS)” (*M* = 2.5, SD = 2.0), and “*online social network sites (e.g., Facebook, Instagram)*” (*M* = 0.5, SD = 1.2) on the above-mentioned response format. The scale showed an acceptable internal consistency across all mentioned friends (*α* = 0.65, *ω* = 0.74; *M* = 1.8, SD = 1.3). The questions on communication behavior were adapted from Greischel, Noack, and Neyer (2016).

#### Friendship quality

To measure perceived friendship quality, we used parts of the Friendship Scale developed by Bukowski et al. (1994). For each of the nominated friends, the respondents answered eight items from two subdimensions (security and closeness, with four items each) on a 4-point Likert scale, ranging from 1 (“*do not agree at all*”) to 4 (“*totally agree*”). Example items included, “*If I have a problem at school or at home, I can talk to my friend about it*”, “*I feel happy, when I’m with my friend*”, and “*Sometimes my friend does things for me, or makes me feel special*”. Confirmatory factor analysis revealed that two items had to be excluded and that the remaining items form one superordinate factor known as “friendship quality”. The scale showed an acceptable internal reliability across all mentioned friends (*α* = 0.75, *ω* = 0.77; *M* = 3.4, *SD* = 0.5).

#### Quality of CMC

Following these loop questions for each of the mentioned friends, we asked the respondents to name the one friend out of their maximum of three mentioned best friends with whom they were writing or talking to most online (on the Internet or via WhatsApp). Regarding their chosen friend (*n* = 41), each respondent answered the following five items adapted from Yarosh, Markopoulos, and Abowd (2014): “I could let < X > know how I was feeling over the Internet”, “I could tell over the Internet how < X > was feeling that day”, “I could tell over the Internet how much < X > cares about me”, “I felt closer to < X > after using the Internet together”, and “Using the Internet with < X > when I was having a bad day helped me feel better”. The items were answered on a four-point Likert scale, ranging from 1 (“*do not agree at all*”) to 4 (“*totally agree*”). Confirmatory factor analysis revealed one superordinate factor, which showed an acceptable internal reliability (*α* = 0.89, *ω* = 0.89; *M* = 2.9, *SD* = 0.8).

#### Control Variables

In addition to the respondents’ age and gender, we also controlled for the nominated friends’ age and gender. In total, 46 percent of the nominated friends were female (*n* = 68). On average, they were 11 years of age (*SD* = 2.1; min = 7; max = 18).

### Data Analysis

In the present study, every respondent was asked to nominate up to three of their best friends. In the main analyses, we investigated characteristics of these friends (level 1 variables) depending on the respondent (level 2 variable). The nominated friends were thus nested within the respondent and their description and evaluation depends on the respondent. Therefore, the friends in this case are not independent units, and the respondent acts as a context within which the mentioned friends are described (Field et al., 2012, p. 858). To consider this hierarchical data structure, we applied multilevel analyses with level 1 and level 2 predictors for the first three research questions (RQ1-RQ3; see Hox, 2010). We calculated various multilevel linear models with FtF-communication with friends, CMC with friends, and perceived friendship quality as respective dependent variables. We applied random intercept and random slope models to consider variations between the respondents. For the multilevel models, *R*^*2*^-values were indicated referring to the explained variance at level 1 and level 2 (see Raudenbush & Bryk, 2002, pp. 74 & 79) as well as the total variance explained (see Snijders & Bosker, 2012, p. 112). To answer RQ4, we filtered for the friend with whom the respondents indicated they wrote or talked with most online. For this analysis, we calculated a multiple linear regressions analysis with quality of online communication as a dependent variable. Missing data were removed listwise for all calculated models.

To analyze the moderating effects of relocation and CMC with friends (RQ2 & RQ3), we stepwise included the two proposed interaction terms (Model 1: no interaction; Model 2: interaction living distance*CMC; Model 3: initiation after relocation*CMC). The models were compared based on Χ^2^ and AIC values. To interpret and illustrate the interaction effects, we referred to Dawson (2014) and used the provided two-way-linear interaction template (available at: www.jeremydawson.co.uk/slopes.htm). For numerical variables, the illustration template uses – by default – one standard deviation above and below the mean to plot the slopes. All analyses were conducted using the software RStudio (Version 1.2.1335) and the lme4 package (Bates et al., 2012).

## Results

### The Role of Residential Relocation for the Communication Behavior with Friends

Initially, we looked at child and adolescent communication behavior with friends, especially after experiencing residential relocation (RQ1, see Table 1). Regarding their FtF-communication with friends, the results confirmed an influence of their experienced relocation. First, we found more FtF-communication with friends who were met after the relocation (*b* = 0.60, *t*(128) = 2.61, *95% CI* [0.15, 1.03]) and lived closer to the respondent (living distance: *b* = −0.43, *t*(132) = −3.88; *95% CI* [−0.64, −0.22]). Furthermore, we also found that child and adolescent FtF-communication (CMC: *b* = 0.22, *t*(135) = 2.26, *95% CI* [0.04, 0.42]) and CMC with friends (FtF-Communication: *b* = 0.17, *t*(130) = 2.50, *95% CI* [0.04, 0.31]) positively reinforced each other. In contrast to their FtF-communication, young users’ CMC was generally more pronounced with older friends (*b* = 0.21, *t*(116) = 2.95, *95% CI* [0.07, 0.35]). Finally, the amount of a respondent’s CMC varied between the mentioned friends (*b* = −0.18, *t*(76) = −2.15, *95% CI* [−0.35, −0.02]), indicating that children and adolescents communicated with some nominated friends more via computer media than with others. For both modes of communication (and more prominently for CMC), more variance could be explained by the respondent him- or herself (level 2; FtF-communication: *R*^*2*^ = 0.28, CMC: *R*^*2*^ = 0.69) than by the respective mentioned friend (level 1; FtF-communication: *R*^*2*^ = 0.21, CMC: *R*^*2*^ = 0.13). In total, 24% for FtF-communication and 49% for CMC could be explained by the integrated variables.

**Table 1:** The role of residential relocation for communication behavior with friends

	FtF-Communication		CMC		Communication via Phone Calls		Communication via Text Messages		Communication via Online Social Network Sites	
*Fixed Effects*	B (SE)	95% CI	B (SE)	95% CI	B (SE)	95% CI	B (SE)	95% CI	B (SE)	95% CI
Age	0.12 (0.10)	[−0.08, 0.31]	**0.16 (0.08)**	[0.00, 0.32]	0.12 (0.13)	[−0.12, 0.36]	0.25 (0.14)	[−0.02, 0.51]	0.10 (0.08)	[−0.05, 0.26]
Gender	−0.23 (0.36)	[−0.93, 0.46]	−0.06 (0.32)	[−0.66, 0.55]	−0.07 (0.48)	[−0.99, 0.86]	−0.04 (0.52)	[−1.03, 0.95]	−0.24 (0.32)	[−0.85, 0.38]
Age Friend	−0.15 (0.09)	[−0.32, 0.02]	**0.21 (0.07)**	[0.07, 0.35]	0.18 (0.11)	[−0.03, 0.39]	**0.32 (0.12)**	[0.10, 0.55]	**0.13 (0.07)**	[0.00, 0.25]
Female Friend	0.11 (0.32)	[−0.51, 0.72]	0.42 (0.27)	[−0.09, 0.94]	0.61 (0.41)	[−0.17, 1.40]	0.26 (0.43)	[−0.56, 1.10]	0.21 (0.25)	[−0.28, 0.70]
Initiation after Relocation	**0.60 (0.23)**	[0.15, 1.03]	−0.21 (0.20)	[−0.59, 0.20]	−0.01 (0.30)	[−0.59, 0.60]	−0.23 (0.32)	[−0.84, 0.40]	−**0.44 (0.19)**	[−0.80, −0.05]
Living Distance	−**0.43 (0.11)**	[−0.64, −0.22]	−0.10 (0.10)	[−0.28, 0.09]	−0.25 (0.15)	[−0.53, 0.03]	0.11 (0.15)	[−0.19, 0.41]	−0.24 (0.16)	[−0.56, 0.08]
FtF-Communication	–	–	**0.17 (0.07)**	[0.04, 0.31]	**0.22 (0.11)**	[0.02, 0.43]	**0.26 (0.11)**	[0.04, 0.49]	−0.02 (0.06)	[−0.10, 0.15]
CMC	**0.22 (0.10)**	[0.04, 0.42]	–	–	–	–	–	–	–	–
Friend	0.09 (0.10)	[−0.10, 0.28]	−**0.18 (0.08)**	[−0.35, −0.02]	−**0.49 (0.13)**	[−0.74, −0.23]	−0.18 (0.13)	[−0.43, 0.07]	0.15 (0.08)	[−0.02, 0.31]
*Random Effects*	SD	95% CI	SD	95% CI	SD	95% CI	SD	95% CI	SD	95% CI
ID/Intercept	**0.73**	[0.35, 0.93]	0.57	[0.00, 0.81]	0.77	[0.00, 1.14]	**1.17**	[0.72, 1.48]	**0.57**	[0.11, 0.81]
ID/Friend	0.00	[0.00, 0.28]	0.21	[0.00, 0.35]	0.37	[0.00, 0.58]	0.00	[0.00, 0.39]	**0.33**	[0.20, 0.45]

*n 14*6; for fixed effects, *B* Unstandardized coefficients, *SE* Standard errors, and the 95% confidence intervals (95% CI) are indicated; for random effects, *SD* Standard deviation and the 95% confidence intervals (95% CI) are indicated; significant effects are marked bold

Although child and adolescent CMC with friends was not influenced by their relocation experiences, a disaggregated look revealed differences between the various forms of CMC (see Table 1). In contrast to phone calls (*M* = 2.4, *SD* = 1.7) and text messages (*M* = 2.5, *SD* = 2.0), communication via online social network sites (*M* = 0.5, SD = 1.2) was evidently less common among the respondents and their close friends. Communication via phone calls (*b* = 0.23, *t*(128) = 2.11, *95% CI* [0.02, 0.43]) and text messages (*B* = 0.26, *t*(135) = 2.28, *95% CI* [0.04, 0.49]) was more pronounced among friends whom respondents also communicated with FtF. Text messages via smartphone were also more common with older friends (*b* = 0.32, *t*(118) = 2.74, *95% CI* [0.10, 0.55]). Additionally, the frequency of making phone calls differed considerably from friend to friend, but it was most pronounced with the closest chosen friend (*b* = −0.49, *t*(78) = −3.67, *95% CI* [−0.74, −0.23]).

Phone calls and text messages with friends were not influenced by relocation experiences. This was different for youngsters’ communication via online social network sites This was stronger with friends users knew prior to the relocation (friendship initiation: *b* = −0.44, *t*(125) = -2.30, *95% CI* [-0.80, -0.05]). This form of CMC was also more frequently used with older friends (*b* = 0.13, *t*(105) = 1.98, *95% CI* [0.00, 0.25]). For all three modes of CMC, more variance could be explained by the respondents (level 2; phone calls: *R*^*2*^ = 0.53, text messages: *R*^*2*^ = 0.53, social networks: *R*^*2*^ = 0.58) than by the mentioned friend (level 1; phone calls: *R*^*2*^ = 0.24, text messages: *R*^*2*^ = −0.04, social networks: *R*^*2*^ = 0.30). In total, a significant amount of variance (phone calls: *R*^*2*^ = 0.37, text messages: *R*^*2*^ = 0.36, social networks: *R*^*2*^ = 0.47) could be explained by the integrated variables.

Ftf-communication with friends (*SD* = 0.73, *95% CI* [0.35, 0.93]) as well as communication via text messages (*SD* = 1.17, *95% CI* [0.71, 1.48]) and social networks (*SD* = 0.57, *95% CI* [0.11, 0.81]) showed significant variance in intercepts across the respondents (see Table 1). In addition, the slopes for communication via social networks varied across the respondents (*SD* = 0.33, *95% CI* [0.20, 0.45]), indicating that the amount of social network communication with the mentioned friends (friend1, friend2, and friend3) varied significantly among the children and adolescents. This means that there is no comparable pattern across the respondents, for example showing most social network communication with the first-mentioned friend and least social network communication with the third mentioned friend.

### The Role of Relocation & Communication Behavior for Perceived Friendship Quality

We further investigated how friendship-related relocation experiences influence child and adolescent perceived friendship quality and if these relationships depend on their friend-related communication behavior (RQ2 & RQ3). Since previous analyses have confirmed that communication via social networks might be the most varying tool when it comes to friendships after relocation (see Table 1), we exclusively focused on this mode of CMC. For the analyses, we created a dichotomized variable, differentiating between those who never use social networks for their communication with friends (*n* = 119; 80%) and those who at least seldom use social networks to communicate with their friends (*n* = 30, 20%).

Regarding pre-existing friendships, it can be assumed that youngsters’ residential relocation can potentially influence friendship quality in a negative manner. This is due to the possible impediment of direct, frequent communication (see Vangelisti, 2015). In line with this assumption, we found a direct negative effect of friends’ living distance on perceived friendship quality (*b* = −0.11, *t*(103) = −3.11, *95% CI* [−0.18, −0.04]; see Table 2, Model 1). Nevertheless, the results also revealed a higher perceived friendship quality for friendships that had previously existed prior to relocation (friendship initiation: *b* = −0.16, *t*(126) = −2.03, *95% CI* [−0.31, −0.01]). The perceived friendship quality was higher for the first mentioned friends (*b* = −0.07, *t*(73) = −2.28, *95% CI* [−0.13, −0.01]), but it generally did not differ according to the indicated friend-related communication behavior; this stood true for communication via FtF as well as social networks (see Table 2).

**Table 2:** The role of residential relocation & communication behavior for perceived friendship quality

	Model 1		Model 2		Model 3	
	*B* (SE)	95% CI	*B* (SE)	95% CI	*B* (SE)	95% CI
*Fixed Effects*						
Age	−0.04 (0.04)	[−0.12, 0.03]	−0.04 (0.04)	[−0.11, 0.03]	−0.05 (0.04)	[−0.12, 0.03]
Gender	−0.14 (0.15)	[−0.42, 0.14]	−0.15 (0.15)	[−0.43, 0.13]	−0.15 (0.14)	[−0.43, 0.13]
Age Friend	0.03 (0.03)	[−0.03, 0.08]	0.02 (0.03)	[−0.03, 0.07]	0.03 (0.03)	[−0.02, 0.08]
Female Friend	0.19 (0.11)	[−0.02, 0.40]	0.15 (0.11)	[−0.05, 0.36]	0.14 (0.10)	[−0.06, 0.34]
Initiation after Relocation	−**0.16 (0.08)**	[−0.31, −0.01]	−**0.16 (0.08)**	[−0.31, −0.01]	−0.06 (0.09)	[−0.23, 0.11]
Living Distance	−**0.11 (0.04)**	[−0.18, −0.04]	−0.07 (0.04)	[−0.15, 0.01]	−0.05 (0.04)	[−0.13, 0.03]
FtF-Communication	−0.02 (0.03)	[−0.07, 0.04]	−0.00 (0.03)	[−0.05, 0.05]	0.00 (0.03)	[−0.05, 0.05]
Communication via social networks	−0.09 (0.11)	[−0.29, 0.11]	0.42 (0.28)	[−0.11, 0.94]	**0.65 (0.29)**	[0.09, 1.20]
Friend	−**0.07 (0.03)**	[−0.13, −0.01]	−**0.06 (0.03)**	[−0.12, −0.00]	−0.05 (0.03)	[−0.11, 0.01]
Living Distance * Communication via social networks	–	–	−**0.16 (0.08)**	[−0.31, −0.01]	−**0.19 (0.08)**	[−0.34, −0.04]
Initiation after Relocation * Communication via social networks	–	–	–	–	−**0.33 (0.16)**	[−0.64, −0.03]
*Random Effects*	SD	95% CI	SD	95% CI	SD	95% CI
ID/Intercept	**0.34**	[0.23, 0.43]	**0.34**	[0.23, 0.43]	**0.36**	[0.25, 0.45]
ID/Friend	0.09	[0.00, 0.14]	0.09	[0.00, 0.14]	0.08	[0.00, 0.13]

*n* 146; for fixed effects, *B* Unstandardized coefficients, *SE* Standard errors, and the 95% confidence intervals (95% CI) are indicated; for random effects, *SD* Standard deviation and the 95% confidence intervals (95% CI) are indicated; significant effects are marked bold

Looking at the random effects, we found that perceived friendship quality (*SD* = 0.36, *95% CI* [0.25, 0.45]) showed significant variance in intercepts across the respondents (see Table 2). In contrast, the slopes for friendship quality across the different mentioned friends did not significantly vary across the respondents, indicating comparable patterns of quality estimations for friend 1, friend 2, and friend 3.

A stepwise inclusion of the two interaction terms between living distance and communication via social networks respectively, friendship initiation and communication via social networks showed a significant improvement of the most complex model 3 (including both interaction terms) compared to model 2 (with only one interaction term, *χ*^*2*^ = 4.55, *p* = 0.033, ΔAIC = −2.55) and model 1 (without the interaction terms, *χ*^*2*^ = 8.64, *p* = 0.013, ΔAIC = −4.64). We, therefore, report the results of the final model 3 including both interaction terms.

**Fig. 1:**
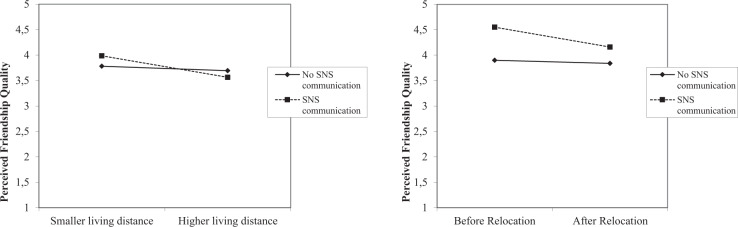
The moderating role of friends‘ communication via social networks for the relationship between living distance (left), friendship initiation (right) and perceived friendship quality

First, we found a significant negative influence from the interaction term regarding living distance and communication via social networks on perceived friendship quality (*b* = −0.19, *t*(101) = −2.38, *95% CI* [−0.34, −0.04]). Looking at this relationship in more detail (see Fig. 1), we observed a slightly higher perceived friendship quality for those friends with whom the respondents also communicated via social networks. This was only true, however, if the living distance between both was rather small. With increasing living distance, the results showed a slight decrease in friendship quality for those who also communicated with their friends via social networks. There was no apparent decrease in friendship quality depending on living distance for friends with whom the respondents did not communicate via social networks.

Second, we also found a significant, negative effect from the interaction term regarding friendship initiation and communication via social networks on perceived friendship quality (*b* = −0.33, *t*(111) = −2.10, *95% CI* [−0.64, −0.03]). Figure 1 illustrates that respondents generally reported a higher friendship quality for the friends with whom they also communicated via social networks, as compared to those with whom they never communicated via social networks. This difference was perceptibly more pronounced when the respective friend was already met prior to residential relocation. Accordingly, it can be assumed that specific forms of CMC can absorb limitations in existing close social relationships after experiencing residential relocation (RQ2). Furthermore, when creating new friendships, a multimodal communication including CMC supports the perceived relationship quality (RQ3).

Regarding perceived friendship quality, more variance could be explained by the respective mentioned friend (level 1; *R2* = 0.33) than by the respondent him- or herself (level 2; *R2* = 0.15). In total, 22% of the perceived friendship quality could be explained by the integrated variables.

Finally, we investigated RQ4 looking at the role of CMC quality on perceived friendship quality. Each respondent answered the questions on CMC quality solely for the friend he or she wrote to or talked with most online (“online friend”, *n* = 41). Controlling for age and gender of the respondents, age and gender of the mentioned “online friends”, and friendship initiation and living distance between friends, we found that only a perceived higher CMC quality predicted a higher overall friendship quality (*b* = 0.30, *95% CI* [0.10, 0.51]). In contrast, neither the frequency of FtF-communication nor the frequency of CMC significantly influenced the relationship quality (see Table 3). Altogether, 36 percent of the variance could be explained by the integrated variables.

**Table 3:** The role of perceived CMC quality for perceived friendship quality

	Perceived Friendship Quality	
	*B* (SE)	95% CI
Age	−0.06 (0.09)	[−0.24, 0.12]
Gender	0.10 (0.23)	[−0.37, 0.58]
Age Friend	−0.06 (0.09)	[−0.25, 0.13]
Female Friend	0.33 (0.27)	[−0.22, 0.87]
Initiation after Relocation	−0.11 (0.14)	[−0.40, 0.17]
Living Distance	−0.16 (0.08)	[−0.33, 0.01]
FtF-Communication	−0.11 (0.06)	[−0.24, 0.01]
CMC	0.04 (0.07)	[−0.11, 0.19]
CMC Quality	**0.30 (0.10)**	[0.10, 0.51]

*n* 14, *B* Unstandardized coefficients, *SE* Standard errors, and the 95% confidence intervals (95% CI) are indicated; significant effects are marked bold

## Discussion

The findings of the present study provide important insights into child and adolescent communication behavior (FtF and computer-mediated) with friends and its relevance for friendship quality after the occurrence of residential relocation. Referring to the socio-ecological framework model by Anderson and colleagues (Anderson et al., 2014a; 2014b), we investigated the influence of residential relocation on FtF-communication and CMC as well as the buffering and reinforcing effects of CMC in the interplay between relocation and friendship quality. Moreover, since quality indicators might be equally – or even more – essential than frequency indicators (Emmers-Sommer, 2004), we additionally looked at the perceived quality of CMC with friends and its role in overall friendship quality.

We could show that the frequency of FtF-communication was higher if the friendship started after the child’s relocation and the living distance was smaller. In contrast, we did not find any effects of relocation on the overall frequency of CMC with friends. Only child and adolescent communication via online social networks was influenced by friendship initiation: children and adolescents reported more communication via social networks with friends they already knew prior to relocation. Hence, youngsters communicated specifically via social networks with well-established friends whom they had known for a longer time. This is in line with previous research confirming that children and adolescents especially use social media to stay in touch and connect with their friends (Lenhart, 2016; Rideout & Robb; 2018, van Driel et al., 2019). In our data, we couldn’t find a significant link between issues concerning relocation and communication via mobile phone (i.e., calls and text messages). From the study, it was apparent that children and adolescents would rather use these modes of communication with friends whom they also met frequently in person. This is in line with research showing that friendships are often cultivated equally, both offline and online (Lenhart, 2016).

Looking at the quality for pre-existing friendships, we can assume that youngsters’ residential relocation may have the potential to negatively influence these relationships (see Vangelisti, 2015). This assumption is supported by the direct negative link between friends’ living distance and their perceived friendship quality within the data. Nonetheless, the reported friendship quality was higher for those friendships that already existed before the relocation. This could simply be due to a longer persistence and history of these friendships that withstood the child’s relocation. Surprisingly, we couldn’t find any significant connection between FtF-communication and friendship quality in the data. On average, the descriptive findings confirmed that the respondents met the mentioned friends several times a week, regardless of whether these friendships were initiated before or after the relocation. This general high amount of personal contact might diminish the role of FtF-communication for the evaluation of friendship quality. Moreover, the quality of FtF-communication may be a more appropriate indicator than frequency (Emmers-Sommer, 2004) and should be implemented in future studies.

Moreover, we found a buffering effect from communication via social networks for existing friendships. If a friend was already met prior to relocation, a higher friendship quality was reported, when both at least seldom also communicated via social networks as opposed to when both never communicated via social networks. This effect also appeared – albeit to a lower extent – for friendships which started after the residential relocation. Thus, CMC may also help to establish and consolidation of new friendships after the occurrence of relocation. This is also in line with the second moderating effect: when living distance between friends was smaller, children and adolescents indicated slightly higher rates of friendship quality with friends whom they also communicated with via social networks. These rates were higher than that of friends with whom they had not communicated in this way. Simultaneously, with increasing living distance, we observed an inverse link indicating slightly higher friendship quality among friends when there was no communication via social networks. Through this, it is evident that a certain degree of spatial closeness is essential for a good friendship, and this precondition of friendships in childhood and adolescence cannot be easily altered by CMC. It can be concluded that communication via social networks is generally used more often with closer friends. Further longitudinal studies are necessary to track whether or not CMC can aid in maintaining a close friendship over the long-term, despite limited personal contact.

The multilevel findings generally confirmed that communication behavior with friends – specifically the level of CMC – depends on characteristics of the respondent more than on the specific mentioned friend. Whether or not an individual communicates with friends via text messages or social networks appears to be a question of a person’s general communication and media use preferences rather than a question of the respective interaction partner.

Moreover, our analyses suggest that the quality of CMC is the determining factor when it comes to friendship quality, not the frequency. In the specific context of residential relocation, the intensity of communication with friends that were left behind potentially decreases. This is due to the fact that the process of orientating and integrating in the new social environment requires an individual’s capacities. However, it can be expected that high-quality contact – albeit more seldom – could possibly buffer potential negative effects of these temporal limitations. Measures on communication quality in the context of CMC are still sparse and need to be further developed and implemented in future research.

Finally, there are some limitations of the present study. First, the present sample was very small, despite drawing from a large-scale, representative survey. Specifically, with the handover from the parent-interview to the child interview, a definite loss of respondents occurred as a result (28%). Relocation with school-age children appeared to be an exception in this case, and moving distance was generally rather small (see also Feijten & van Ham, 2013). The large mean time since relocation within the sample was, on average, 12 months. Through examining this, it is apparent that families are more likely to participate in a study when some time has passed since the stressful event of moving (Anderson et al., 2014a; 2014b). This also means that reorganization of social life in these families has already proceeded, limiting the information regarding direct social cutback experiences. Due to the small sample size, several comparisons (e.g., regarding the preceding duration of relocation or the reasons for relocation) were restricted. However, the main analyses were based on multilevel calculations that referred to a larger sample of nominated friends (*N* = 149; level 1). There seem to be no commonly accepted rules for sample size in multilevel studies, but some authors have previously suggested reference values. Kreft and de Leeuw (1998), for example, indicated that there should be at least 20 contexts – or groups – on level 2 and that group sizes should not be too small when calculating cross-level interactions. The low average number of friends per respondent (2.6) was due to the predefined procedure of our study, and this might especially limit the power for testing between-respondent variances of effects regarding friend-level variables (Snijders, 2005). Second, there were some parents who initially indicated through the filter question that they had moved into another town or village. Afterwards, however, they mentioned that the move occurred within the same town or village. Since our main analyses controlled for the living distance between the children and their friends, and the overall sample size was small, we abstained from deleting these cases. However, a small moving distance also reduces the social reorganization requirements. This needs to be considered when interpreting the study findings. Third, the chosen procedure of mentioning up to three best friends resulted in a selective pool of very close friendships which could generally be characterized by a high amount of quality and contact. This might not be generalizable to all friendships. In future research, it could be promising to differentiate between best friends, good friends, and looser acquaintances in order to detect more differences in communication behavior and its role in friendship quality.

Despite these limitations, these findings can be considered a starting point for more research on the importance of communication behavior (FtF and computer-mediated) for the relationship quality of children and adolescents, especially in specific and stressful living situations. This is also relevant and applicable to the current COVID-19 pandemic. Children and young people were not able to meet their friends in person during the strict stay-at-home orders. Studies have shown that the exchange with friends was helpful for coping with the challenges and aided against loneliness, especially during those times (e.g., Sun et al., 2020). For adolescents, digital media offered a good alternative to FtF-meetings (Langmeyer et al., 2020). The present study also confirmed that digital media are a good way to stay in touch with friends, but they cannot replace FtF-interactions. This is why it is vital that – as much as is possible – children and young people are presented with opportunities to meet their friends in person.

Future studies should rely on qualitative data to gain in-depth insights into the motives, dynamics, and quality of children’s everyday communication with various reference persons. On the other hand, innovative methods, such as mobile experience sampling, can help to investigate the situational components associated with interpersonal communication. This enables a more detailed analysis on the use of various communication channels and possibilities with persons who – socially and spatially – may be more close or more distant, and on the respective communication quality as well.
